# Anti-obesity carbonic anhydrase inhibitors: challenges and opportunities

**DOI:** 10.1080/14756366.2022.2121393

**Published:** 2022-09-08

**Authors:** Claudiu T. Supuran

**Affiliations:** NEUROFARBA Department, Sezione di Scienze Farmaceutiche e Nutraceutiche, Università degli Studi di Firenze, Firenze, Italy

**Keywords:** Carbonic anhydrase, mitochondria, de novo lipogenesis, fatty acid biosynthesis, obesity, topiramate, zonisamide, sulphonamides, phenols, natural products

## Abstract

The mitochondrial isoforms VA/VB of metalloenzyme carbonic anhydrase (CA, EC 4.2.1.1) are involved in metabolic processes, such as *de novo* lipogenesis and fatty acid biosynthesis. We review the drug design landscape for obtaining CA VA/VB-selective/effective inhibitors, starting from the clinical observations that CA inhibitory drugs, such as the antiepileptics topiramate and zonisamide, or the diuretic acetazolamide induce a significant weight loss. The main approaches for designing such compounds consisted in drug repurposing of already known CA inhibitors (CAIs); screening of synthetic/natural products libraries both in the classical and virtual modes, and *de novo* drug design using the tail approach. A number of such studies allowed the identification of lead compounds diverse from sulphonamides, such as tropolones, phenols, polyphenols, flavones, glycosides, fludarabine, lenvatinib, rufinamide, etc., for which the binding mode to the enzyme is not always well understood. Classical drug design studies of sulphonamides, sulfamates and sulfamides afforded low nanomolar mitochondrial CA-selective inhibitors, but detailed antiobesity studies were poorly performed with most of them. A breakthrough in the field may be constituted by the design of hybrids incorporating CAIs and other antiobesity chemotypes.

## 1. Introduction

Obesity, a condition characterised by excessive fat accumulation, with body mass indexes (BMIs) ≥ 30 kg/m^2^ constitutes nowadays a challenging medical problem worldwide, with a large number of affected people both in the developed and developing countries. Only in USA it is estimated that 2/3 of the population has body weight problems, with 1 each three adults and 20% of the adolescents being obese^1^^,^^2^. Obesity is a multifactorial, complex medical problem, being considered nowadays as a chronic, degenerative disease^3^ which however, has a large number of metabolic and psychological comorbidities, making it difficult to manage/treat, both medically and socially^1–3^. Some of these comorbidities include metabolic dysfunctions (type 2 diabetes, fatty liver diseases, dyslipidemia, gallstones, gout)^1^^,^^2^; cardiovascular diseases (atherosclerosis, hypertension, atrial fibrillation, heart failure); enhanced possibility of getting cancer (among which colon, breast and pancreas tumours); pulmonary problems (sleep apnoea, asthma); mental problems (cognition deficit, depression, anxiety and panic disorders)^1–3^ and many other inconveniences which are not mentioned in detail here, as the field was recently reviewed in at least two excellent papers^1^^,^^2^. Apart a proper and balanced (hypocaloric) diet^4^, and bariatric surgery^5^, the pharmacological armamentarium for the treatment of obesity is rather scarce, with few available efficient drugs, which in addition possess a large number of side effects^1^^,^^2^^,^^6^. Furthermore, there is a rather relevant number of antiobesity drugs which have been developed, approved and withdrawn after a short period (few months – one year) due to serious side effects that emerged after their use in a relatively consistent number of patients, immediately after their approval^1^^,^^2^.

As already mentioned, the field of antiobesity drugs and the relevant drug design strategies to obtain them were recently reviewed^1^^,^^2^, and for this reason the physio-pathological processes involved in obesity as well as the various drug targets which have been explored in the last decades and which led to the few clinically used agents will be not dealt with here. The scope of this article is to review recent devlopments in the drug design of inhibitors of carbonic anhydrases (CAs), rather orphan anti-obesity drug targets^7^, which however may lead to interesting developments, as already shown in previous reviews on this argument, the latest of which was published in 2013^8–12^.

## 2. Carbonic anhydrases role(s) in obesity

CAs (EC 4.2.1.1) are enzymes which catalyse the interconversion between CO_2_ and bicarbonate, thus also generating a proton, and thus deeply involved in pH regulation^13^^,^^14^. Widespread in organisms all over the phylogenetic tree, from Archaea, Bacteria to Eukaryotes, at least 8 genetic families encoding CAs are known to date, of which only α-CAs are present in vertebrates, including humans^13–15^. Fifteen human (h) CA isoforms, hCA I-hCA XIV (with two V-type ones, VA and VB) were described and characterised in detail, with many of them being consolidated drug targets for obtaining diuretics, antiglaucoma, antiepileptic and antitumor agents, among others^13–15^.

**Figure 1.**
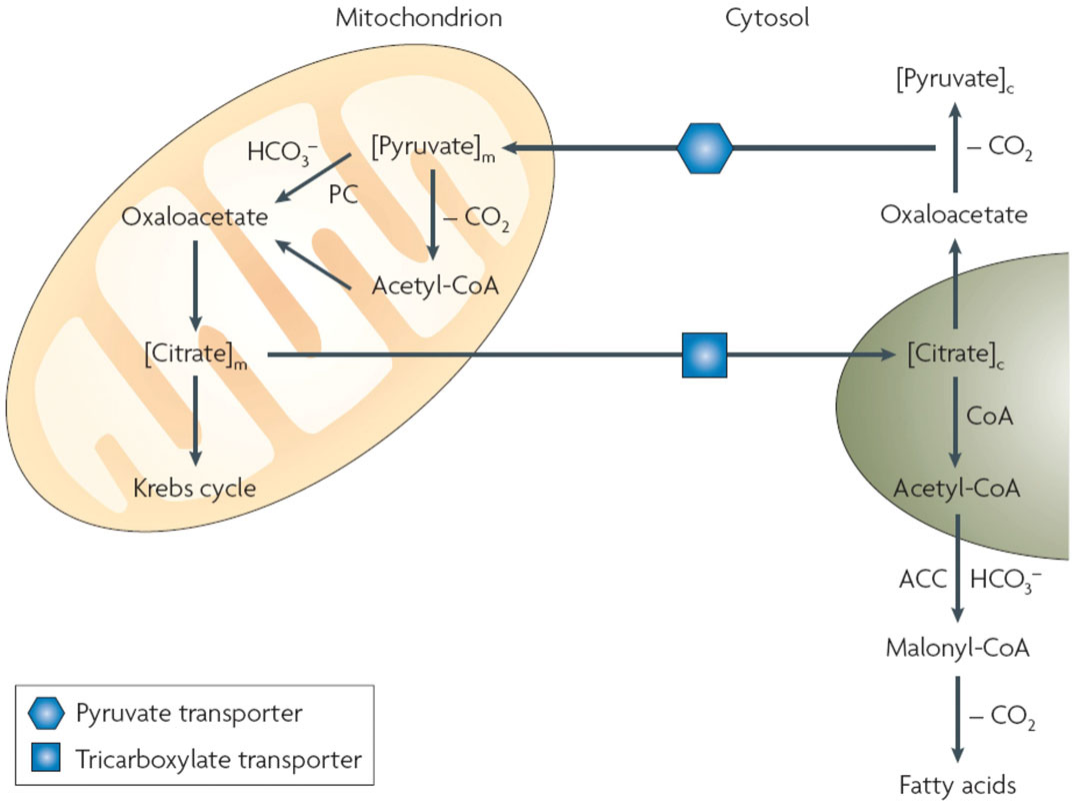
Role of mitochondrial and cytosolic CA isoforms in fatty acid biosynthesis: the transfer of acetyl groups from the mitochondrion to the cytosol (in the form of citrate) is required for the provision of substrate for *de novo* lipogenesis via malonyl-coenzyme A as key intermediate. Steps involving bicarbonate, both for the reaction catalysed by pyruvate carboxylase (PC) and acetyl-coenzyme A carboxylase (ACC) require the presence of CA isozymes: CA VA/VB in the mitochondrion and CA II in the cytosol.

In the last period, CAs were not only considered as being involved in pH regulation/buffering in many cells and tissues, but also as metabolic enzymes^16^, due to their demonstrated role in several metabolic processes in tumours^17^^,^^18^ and normal cells, including fatty acid biosynthesis and *de novo* lipogenesis (DNL) – Figure 1^2^^,^^8–12^. It has been known for decades that fatty acid biosynthesis and DNL involve both mitochondrial and cytosolic steps, in which several enzymes implicated both in the Krebs cycle as well as DNL, among which pyruvate carboxylase (PC) and acetyl-coenzyme A carboxylase (ACC) use bicarbonate and not CO_2_ as one of their substrate^8–12^. In order to achieve the very rapid interconversion between these two species, highly catalytically active CA isoforms (among which CA II in the cytosol^13–15^ and CA VA/VB in the mitochondria^19^^,^^20^) are necessary to participate^21–25^. It has been demonstrated already in the 90 s that this is indeed the case, and that inhibition of mitochondrial/cytosolic CAs interferes with fatty acid biosynthesis and DNL in various cells, tissues and animal models^21–25^.

The most detailed study regarding the role of mitochondrial CAs in metabolism was reported by Minteer’s group in 2013^26^ by using mitochondria wired onto electrodes and selective mitochondrial CA inhibitors (CAIs) – see discussion later in the text. The metabolism of pyruvate, acetate, and succinate were examined, in the presence of specific CA VA/B inhibitors, by measuring metabolic energy conversion, and comparing the resulting metabolic differences after treatment with structurally diverse, but effective CAIs of the primary sulphonamide type. It has been thus observed that some CA VA/B inhibitors showed a broad spectrum inhibition of metabolism, where others only had significant effects on some metabolic pathways, with pyruvate metabolism being the most dramatically affected by CA inhibition, followed by fatty acid metabolism, and finally by succinate metabolism^26^. These data conclusively demonstrated a clear role of mitochondrial CAs in metabolism and fatty acid biosynthesis, but the idea to use inhibition of such enzymes for obtaining antiobesity drugs started to be considered only in 2000s, when a drug company now no longer existing (Solvay Pharmaceuticals) and some academic research groups started a program for obtaining CAIs with antiobesity activity^27^. It should be noted, that as with other CA-related fields, such as CAIs as antitumor agents or anti-infectives, there is a serious “resistance” from members of the scientific community to accept a role of CAs in metabolism and obesity, with many detractors trying to interfere with these findings by obstructing their publication, performing dishonest reviewing of manuscripts dealing with this topic and similar activities, which will however not stop a field which revealed to be an innovative one, with potential significant benefits for many patients suffering from this disease.

It should also be mentioned that there were many reports on the possible role of another CA isoform, the cytosolic CA III, in lipogenesis and obesity^28–30^. However, it was recently demonstrated that CA III is not involved in lipogenesis^31^, and we will not discuss this isoform as a possible antiobesity drug target here, also considering its very low catalytic activity for the CO_2_ conversion reaction to bicarbonate as well as low affinity for sulphonamide/sulfamate inhibitors^32^.

## 3. Drug design of CA inhibitors as antiobesity agents

Although the CAI drug design panorama is quite rich, with many strategies and studies reported for applications as different as antiglaucoma, anticonvulsant, antitumor, anti-neuropathic pain and antiinfective agents^13^^,^^33–35^, the field of antiobesity CAIs was less investigated. However, three main approaches may be envisaged at this moment, which produced several interesting developments over the last two decades: (i) repurposing of drugs originally discovered for other pharmacologic applications than obesity; (ii) screening of natural products/synthetic libraries by using either virtual screening procedures or more classical enzyme inhibition assays; and (iii) *de novo* drug design studies based on already identified leads or on structural biology data of enzyme-inhibiytor adducts characterised in detail, mainly by X-ray crystallographic techniques^13^^,^^14^^,^^33–35^.

### 3.1. Drug repurposing

**Figure 2.**
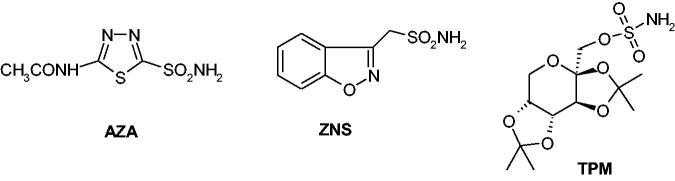
Sulphonamide/sulfamate CAIs in clinical use: acetazolamide (**AAZ**), zonisamide (**ZNS**) and topiramate (**TPM**).

Sulphonamides and their isosteres (sulfamates, sulfamides) are the most investigated CAIs^13^, with many representatives in clinical use for decades as diuretics, antiglaucoma, antiepileptic, and even antiinfective agents, whereas many other such derivatives are in clinical trials for novel applications^13^^,^^14^^,^^33–35^. Sulphonamides such as acetazolamide (**AAZ**), launched in 1954 as the first non-mercurial diuretic^13^, zonisamide (**ZNS**) or the sugar sulfamate derivative topiramate (**TPM**) – Figure 2, are well known CAIs, with the last two compounds originally reported as antiepileptic agents^13^^,^^36^^,^^37^ but later shown to induce a significant weight loss, both in animal models^38^, clinically controlled trials^39–42^ and anecdotal reports^43–48^.

Thus, the use of **AZA, ZNS** and **TPM** as anti-obesity drugs may be considered an interesting, sucessful but also problematic example of drug repurposing, since the use of these CAIs alone or in combination with other agents (phentermine, bupropion, metformin)^49^^,^^50^ was demonstrated to induce weight loss in many obese patients, also improving their blood glucose levels^39–50^. How do these agents exert their antiobesity beneficial effects? Although the pharmacology of **TPM** and **ZNS** is rather complex, as these compounds bind to a multitude of targets^27^^,^^36^, both of them and obviously **AAZ**, are effective CAIs against human (h) CA (hCA) isoforms involved in fatty acid biosynthesis/DNL^13^^,^^51^^,^^52^ – Table 1.

**Table 1.** CA inhibition data with selected drugs/investigational compounds against human (h) isoforms hCA I, II, VA and VB^13^^,^^51^^,^^52^.

	K_I_ (nM)				
Drug/Cmpnd	hCA I	hCA II	hCA VA	hCA VB	Ref
**AAZ**	250	12	63	54	13
**ZNS**	56	35	20	6033	52
**TPM**	250	10	63	30	51

**Figure 3.**
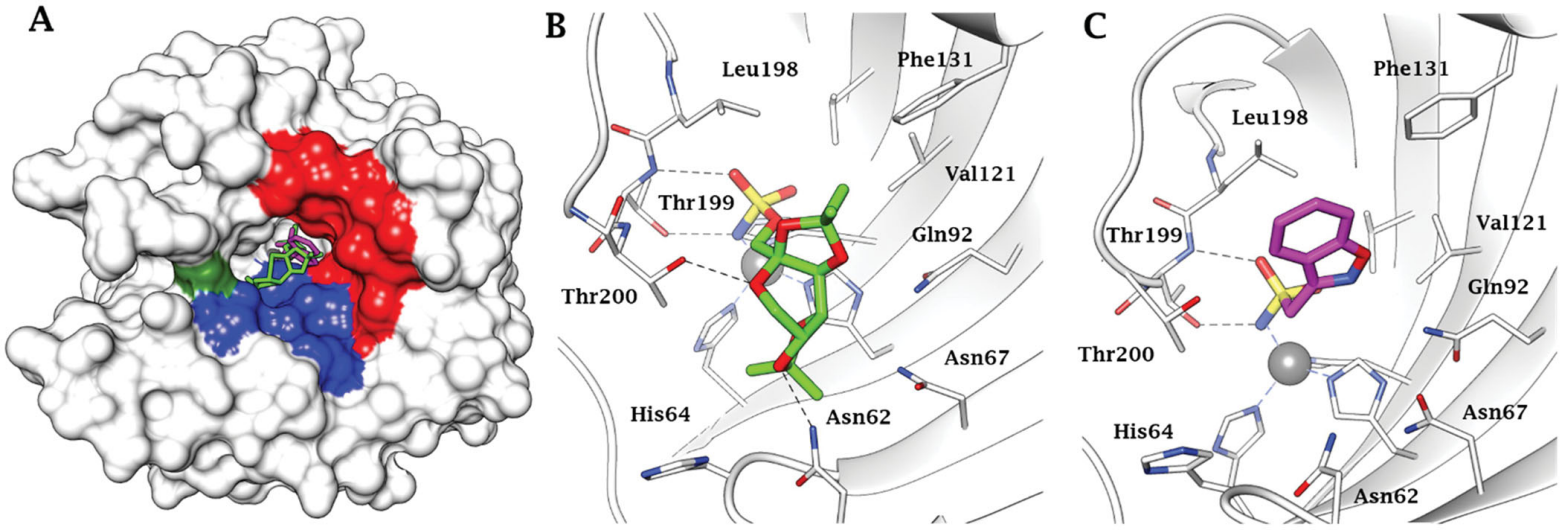
(A) hCA II complexed with superimposed topiramate (PDB 3HKU) in green, and zonisamide (PDB not deposited, available from the authors^52^) in magenta. The Zn(II) is shown as a grey sphere that is bound to the protein ligands His94, His96 and His119. The hydrophobic half of the active site is coloured in red, the hydrophilic one in blue. His64, the proton shuttle residue, in green. Active site ribbon view of hCA II in adduct with B) **TPM** and C) **ZNS**. H-bonds are represented as black dashed lines. Active site ribbon view of hCA II in adduct with B) topiramate and C) zonisamide. H-bonds are represented as black dashed lines.

From data of Table 1 it may be seen that all three drugs are effective hCA II, hCA VA and hCA VB inhibitors (inhibition constants of 10–63 nM) except **ZNS** against hCA VB, case in which the inhibition constant was in the micromolar range^52^. Furthermore, the binding of the two antiepileptics has also been investigated by X-ray crystallography on isoform hCA II^51^^,^^52^ (as the X-ray crystal structure of hCA VA is not known at this moment^53^) and as seen in Figure 3, interesting findings emerged. **TPM** binds towards the hydrophilic half of the active site whereas **ZNS** scaffold is orientated more towards the hydrophobic half (Figure 3(A)), but both compounds coordinate through their zinc binding group (ZBG) in deprotonated form as sulfamate and sulfonamidate, respectively, to the catalytic metal ion, substituting the water molecule/hydroxide ion which acts as nucleophile in the catalytic cycle^54^ – Figure 3(B,C). In the case of topiramate, the sugar scaffold makes a rather large number of H-bonds which involve the oxygen atoms from the drug and hydrophilic amino acid residues from the active site, among which Thr199, Thr200, Asn62, Asn67 and Gln92 (Figure 3(B)), which strongly stabilise the enzyme-inhibitor complex (K_I_ of 10 nM). In the case of **ZNS** adduct, except the ZBG which participates in H-bonds with Thr199, the scaffold of the inhibitor participates only in van der Waals interactions with hydrophobic residues, among which Val121, Phe131 and Leu198 (Figure 3(C)). Although the two compounds show such a diverse binding mode to the enzyme, their inhibitory power towards hCA II is comparable (and also similar to that of **AAZ**, which as all sulphonamides, binds deeply within the active site, coordinating to the zinc ion though the SO_2_NH^-^ moiety. The binding of **AAZ** was discussed in detail in many previous article, see refs.^33^^,^^35^^,^^54^) Although the detailed binding of **TPM** and **ZNS** to hCA II (and in the case of **TPM** also to hCA I^55^) are well understood, the two compounds were not used as leads for obtaining more efficient antiobesity CAIs, probably due to the difficulty to derivatize. Indeed, the only study in which the sulfamate ZBG of **TPM** was changed to sulfamide, led to compounds with decreased efficiency as CAIs^56^. It should be however mentioned that the binding of **ZNS** and **TPM** to hCA VA has been investigated by computational techniques, which explained the efficient binding to the enzyme and the differences in inhibitory power towards the mitochondrial and cytosolic isoforms^57^.

### 3.2. Screening of natural products/synthetic libraries

**Figure 4.**
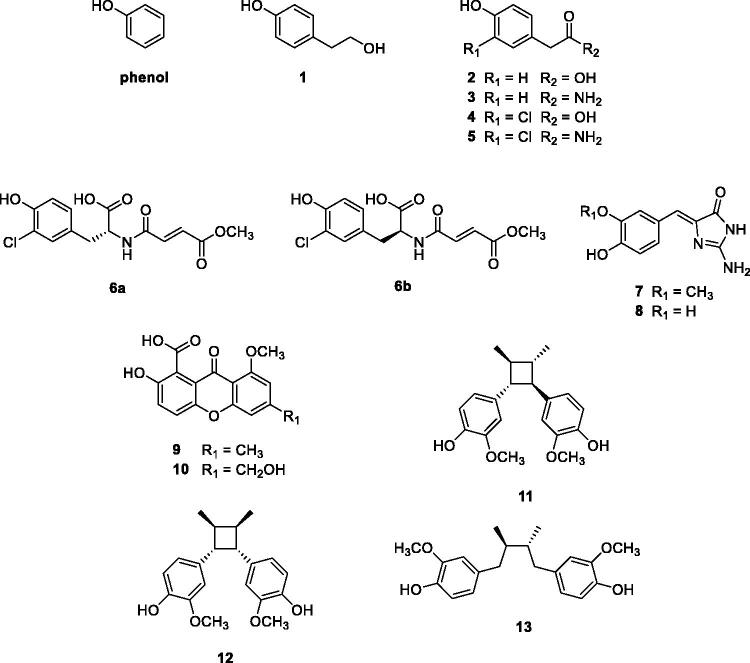
Phenol and NP based phenols screened as hCA VA/VB inhibitors.

The first screening study based on a library of natural-based phenols for the identifiction of hCA VA and VB inhibitors was reported by Davis et al.^58^. Phenol is indeed a weak CAI, which binds differently from the sulphonamides, as its OH moiety is anchored to the zinc-coordinated water molecule^54^. Using phenol as lead, a library of simple and more complex natural product (NP) phenols of types **1–13** have been screened (Figure 4) as inhibitors of hCA I, II (as offtargets) and hCA VA and VB (as target enzymes). Many of these derivatives were micromolar hCA I and II inhibitors, whereas acting more effectively as inhibitors of hCA VA/VB, with K_I_s in the range of 90–105 nM^58^.

**Figure 5.**
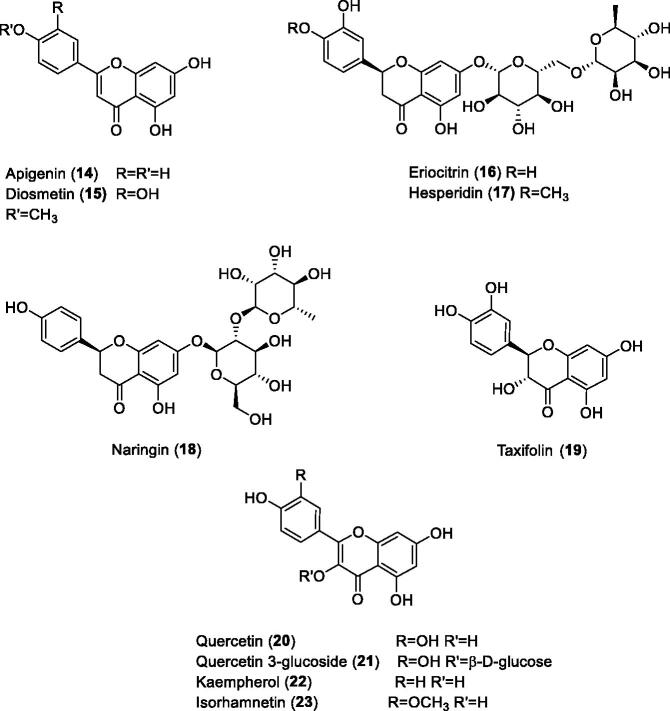
NPs screened as hCA VA/VB inhibitors.

Gidaro et al.^59^ screened a library of NPs including polyphenols, flavones and some of their glycosides, for the inhibition of several CA isoforms, including hCA VA (Figure 5). Many of these derivatives acted as low micromolar hCA VA inhibitors (but they also inhibited isoforms hCA I, IX and XII), and the most effective mitochondrial CA inhibitors were detected to be apigenin **14** (K_I_ of 0.30 µM) and eriocitrin **16** (K_I_ of 0.15 µM). In the same work a computational study has been performed for the binding of **16** to murine (m) mCA VA (for which a truncated X-ray crystal structure is available^54^) which led to the proposal that this flavone coordinates with its catecholic system to the zinc ion from the enzyme active site^59^. This binding mode is however not much plausible, considering that catechols were recently shown by X-ray crystallography to anchor to the zinc-coordinated water and to the deep water molecule within the active site of hCA II^60^a. Some natural product polyphenols isolated from blueberries and their glycosides were also investigated as mitochondrial CA inhibitors and showed micromolar inhibitory action^60^b.

In another study, Costa et al.^61^ examined NP present in essential oils isolated from natural sources, of types **24–33** for the inhibition of hCA I, II and VA (Table 2). The tropolone **24** was thus reported as a new chemotype with CA inhibitory properties, being proposed (by using computational data) that it coordinates bidentately with its CO-CHOH moiety to the zinc ion from the enzyme active site. In this case, X-ray crystallography of a simple tropolone confirmed the proposed binding mode^62^. 2-Hydroxyisobutyric acid **25** and 3Z-nonenoic acid **29** were the most effective hCA VA inhibitors detected in that study, with K_I_s < 5 µM and also showing selectivity for inhibiting hCA VA over the dominant and widespread offtarget isoform hCA II^61^.

**Table 2.** Structures of compounds **24**–**33** screened for the inhibition of hCA I, II and VA.

Compound		K_I_ (μM)		
		hCA I	hCA II	hCA VA
**24**	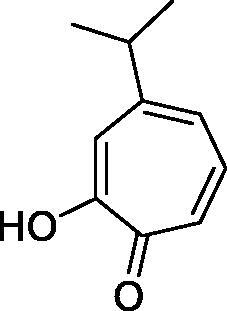	4.98	90.60	7.50
**25**	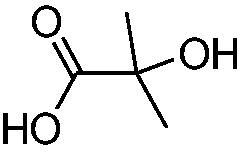	5.18	>100	3.80
**26**	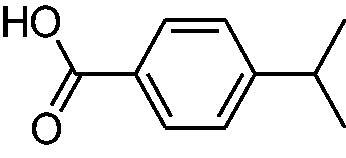	3.44	85.40	7.38
**27**	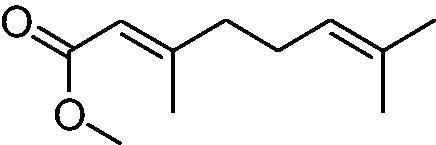	8.27	>100	8.36
**28**	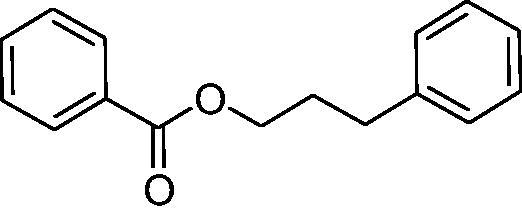	3.75	>100	7.83
**29**	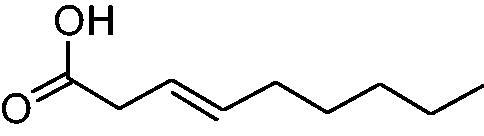	6.09	>100	4.52
**30**	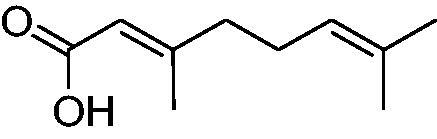	8.81	18.90	9.07
**31**	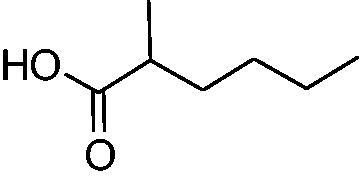	5.06	3.71	43.10
**32**	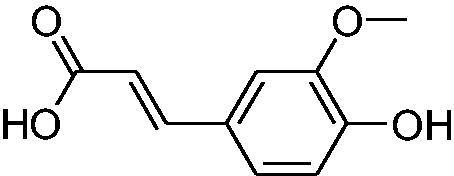	6.82	>100	9.69
**33**	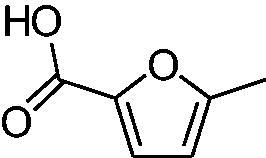	4.89	>100	44.00

The traditional names of the compounds are: **24:** β-thujaplicin; **25**: 2-hydroxyisobutyric acid; **26:** 4-isopropylbenzoic acid; **27**: methyl geranate; **28:** 3-phenylpropyl benzoate; **29:** 3Z-nonenoic acid; **30:** Z-geranic acid; **31:** 2-methylhexanoic acid; **32**: ferulic acid; **33**: 5-methylfuran-2-carboxylic acid.

**Figure 6.**
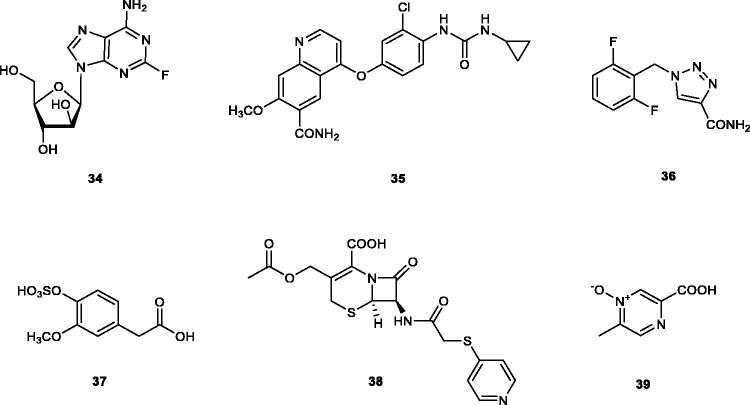
hCA VA inhibitors **34–38** identified by VS techniques, and acipimox **39**, identified by classical screening procedures.

Virtual screening (VS) procedures were also applied by Alcaro’s group^63^^,^^64^ in order to detect antiobesity compounds based on the inhibition of hCA VA. A library of 93522 compounds was used in a VS procedure, which led to 12 hits, which were therafter tested experimentally for inhibition of hCA I, II and VA^63^. Among the obtained hits were the anticancer drugs fludarabine **34**, lenvatinib **35**, the antiepileptic rufinamide **36** (micromolar inhibitors, K_I_s of 130–344 nM), as well as the homovanillic acid sulphate **37** and the desacetyl metabolite of the antibacterial cephapirin **38**, which were active in the nanomolar range against hCA VA, with no inhibition at all of hCA I and II (Figure 6).

These compounds were docked within the murine (m) enzyme mCA VA active site in the same study, but the binding poses obtained were not yet validated by X-ray crystallography. However, they may provide interesting hints for the drug design of novel CA VA inhibitors belonging to new chemotypes compared to the well known sulphonamides, sulfamates or phenol derivatives. Acipimox **39**, a nicotinic acid derivative in clinical use for the treatment of hyperlipidaemia (Figure 6) was also reported recently^65^ to act as low micromolar hCA VA/VB inhibitor. By using computational methods, the same study suggested that acipimox coordinates through its carboxylate moiety to the zinc ion from the CA active site^65^.

### 3.3. De novo drug design

Several studies explored the *de novo* drug design of hCA VA/VB inhibitors, mainly belonging to zinc binders (sulphonamides and their isosteres)^54^ by using the tail approach, which consists in attaching moieties (tails) on the scaffold of the CAIs in such a way as to permit the contact with the more external parts (entrance to the cavity and its rim) of the CA active site, where the amino acid composition between the various isoforms has the highest variability^66^^,^^67^.

One of the first such studies^68^ examined a series of 46 aromatic and heterocyclic sulphonamides, many of which were simple derivatives of benzenesulfonamide and 1,3,4-thiadiazole-5-sulphonamide for the inhibition of mCA VA and hCA I, II and IV (as offtargets). Some of the best inhibitors (in the nanomolar range, typically K_I_-s of 6–95 nM) incorporated the benzenesulfonamide head and acylamido or ureido tails on the sulphanilamide scaffold, which in turn possessed alkyl or aryl moieties. Although this series was not tested for the inhibition of hCA VA/VB (but only the murine isoform VA), the leads detected were useful for the next generation hCA VA/VB inhibitors that were reported thereafter. Indeed, Guzel et al.^69^ reported a large series of aromatic/heterocyclic sulphonamides incorporating phenacetyl, pyridylacetyl and thienylacetyl tails of types **40–44** (Table 3), some of which were among the most effective and isoform V-selective sulphonamides discovered to date.

**Table 3.** Benzenesulfonamides and 1,3,4-thiadiazole-sulphonamides acting as low nanomolar hCA VA and VB inhibitors^69^.

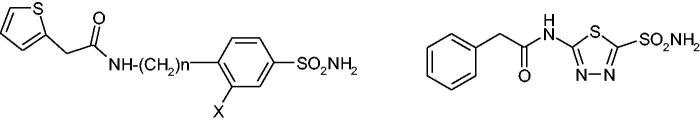				
	40-43		44	
			K_I_ (nM)	
Compound	n	X	hCA VA	hCA VB
**40**	0	–	7.2	7.0
**41**	0	Cl	7.7	8.6
**42**	1	–	9.1	7.2
**43**	2	–	10.2	8.0
**44**	–	–	8.4	6.1

It can be seen that these sulphonamides showed low nM inhibitory action against both mitochondrial isoforms (K_I_s of 6.1 − 10,2 nM) and they were also employed by Arechederra et al.^26^ to investigate the metabolic fluxes in the presence of hCA VA/B inhibitors, as mentioned earlier in this review. Although these sulphonamides also inhibit the cytosolic hCA I and II (data not shown), their K_I_s against these isoforms are at least an order of magnitude higher compared to the same parameters measured for hCA VA/B^69^.

**Figure 7.**
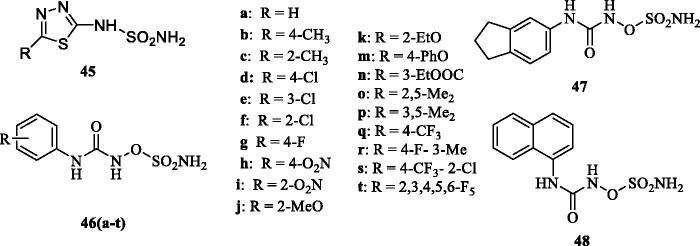
Sulfamides **45** and sulfamates **46–48** reported as hCA VA/VB inhibitors.

Smaine et al.^70^ reported a small series of 2-substituted-1,3,4-thiadiazole-5-sulfamides **45** which showed inhibitory activity against hCA I, II and IV (K_I_s in the high nanomolar to the micromolar range) but were low nanomolar inhibitors of hCA VA/VB, with inhibition constants in the range of 4.2–32 nM and 1.3–74 nM, respectively. The most effective derivatives incorporated as R the groups CF_3_ (K_I_ of 7.3 nM for hCA VA and of 3.9 nM against hCA VB) and 4-bromophenyl (K_I_ of 4.2 nM for hCA VA and of 4.5 nM against hCA VB)^70^ – see Figure 7.

Poli et al.^71^ reported a series of *N*-aryl-*N*'-ureido-*O*-sulfamates of types **46–48** (Figure 7) and tested them against the two mitochondrial CA isoforms hCA VA and VB. The results revealed an interesting selectivity profile, especially against hCA VB over the VA, observed for all the analysed compounds. For one derivative **46o** (K_I_ against hCA VA >10 µM, K_I_ against hCA VB of 515 nM), molecular modelling studies highlighted the importance of amino acid residues which are diverse at the entrance of the cavity between the two mitochondrial isoforms, which substantially influenced the tail orientation of the inhibitor, its interaction with the enzymes and were reflected on the potency of the inhibitor against the mitochondrial CAs^71^.

**Figure 8.**
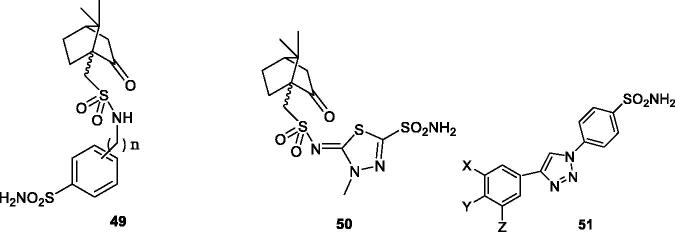
Aromatic/heterocyclic sufonamides **49** and **50** incorporating 10-camphorsulfonyl tails. In derivatives **49**, *n* = 0, 1 and 2. Triazole-sulphonamides **51** incorporate X, Y Z groups of the type H, F, Me, OMe, CF_3_, SO_2_NH_2_.

Maresca and Supuran^72^ reported a series of (R)- and (S)-10-camphorsulfonyl-substituted aromatic/heterocyclic sulphonamides of types **49** and **50** which showed effective (usually low nanomolar efficacy) for inhibiting hCA VA/VB and having less affinity for hCA I and II. The (R) enantiomers were generally more effective as mitochondrial CA inhibitors over the corresponding (S) enantiomers^72^. Poulsen et al.^73^ reported triazole-sulphonamides **51** obtained by click chemistry and incorporating various groups on the second aromatic ring (Figure 8), which showed effective, nanomolar inhibition of hCA VA and VB, but also potently inhibited hCA II, being thus non-selective inhibitors.

## 4. Conclusions and future developments

Starting from the observations that clinically used antiepileptics (topiramate, zonisamide) or diuretics (acetazolamide) induce weight loss in obese patients, as well as reduction of blood glucose levels, a rationale was searched for explaining this effect, which concentrated on the fact that these compounds are effective inhibitors of several CA isoforems, including the mitochondrial ones, hCA VA and VB, known to be involved in DNL and fatty acids biosynthesis. It should be stressed here that these drugs do not induce mitochondrial toxicity as erroneously stated by some reviewers of this manuscript when it has been submitted in another journal to which I refuse to continue to contribute due to conceptual errors and misunderstanding during the reviewing process. Although the two antiepileptic drugs mentioned here possess a complicated polypharmacology, topiramate in combination with phentermine was approved by FDA in 2012 for a second use in the management of obesity, whereas there are clinical trials on the similar use of zonisamide, alone or in combination with other agents (bupropion, metformin)^49^^,^^50^. However, the side effects due to the non-selective inhibition of the target CA isoforms and the polypharmacology of these drugs, makes them effective but scarcely used antiobesity agents.

As a consequence, drug design studies have emerged in the last two decades for the design of potent and isoform-selective hCA VA/VB inhibitors. The main strategies which have been used consisted (in addition to the drug repurposing just mentioned) in the screening (experimentally or by using VS) of large libraries of synthetic and natural products, which afforded interesting and somehow unexpected hints, such as for example the tropolones (a new chemotype with CA inhibitory properties)^61^, several flavones, polyphenols and their glycosides^59^, but also clinically used drugs, such as fludarabine, lenvatinib, rufinamide, etc.^63^. The classical drug design studies for obtaining effective and isoform-selective hCA VA/VB inhibitors concentrated on the other hand on the classical sulphonamide/sulfamate/sulfamide chemotypes^66–73^. However, a rather limited number of such studies has been reported to date, and although several highly effective compounds were detected, detailed pharmacological studies which prove their efficacy in the inhibition of DNL and in the promotion of weight loss in experimental models of obesity, are unfortunately lacking.

The lack of X-ray crystal structures of isoforms hCA VA/VB^53^^,^^54^ was detrimental to the structure-based drug design of inhibitors targeting these isoforms, although several homology models and other computational studies addressed this problem^61^^,^^63^^,^^64^^,^^71^. In some cases^61^ the proposed binding modes of inhibitors such as the tropolones were confirmed by crystallography^62^, whereas in other cases, such as for some catechol natural products^59^ the crystallography^60^ did not confirm the binding poses obtained by computational techniques. One of the detailed such studies, by Tuccinardi’s group^71^ for sulfamate CAIs analysed in detail the selectivity profile of compounds which potently inhibited hCA VB and were weak or ineffective as hCA VA inhibitors, revealing the amino acid residues at the entrance of the active site cavity involved in binding. With all these limitations, the field of CA VA/B inhibitors with potential anti-obesity activity made relevant progress in the last decade since the last review in the field was published^12^. Nowadays there are many classes of such potent and also selective mitochondrial CA inhibitors, apart the sulphonamides and sulfamates reported earlier, which strengthen the rationale of using of topiramate and zonisamide as anti-obesity agents, alone or in combination with other drugs, wirh all their limitaions mentioned above due to side effects correlated or no with offtarget CA inhibition^40–42^.

Although a highly innovative procedure for evaluating the metabolic fluxes in the presence of mitochondrial CA inhibitors has been reported already in 2013^26^, which made use of electrode wired mitochondria, this type of experiments were performed only with a limited number of sulphonamide CAIs, which however, conclusively showed that the pyruvate, succinate and overall cell metabolism undergoes significant changes when these two enzymes are inhibited (without mitochondrial toxicity, I stress this again). This type of measurements would be desirable for other classes of newly identified CA VA/B inhibitors, prior to *in vivo* anti-obesity experiments, in animal models of the disease, which are more expensive, as well as time- and resource consuming.

**Figure 9.**
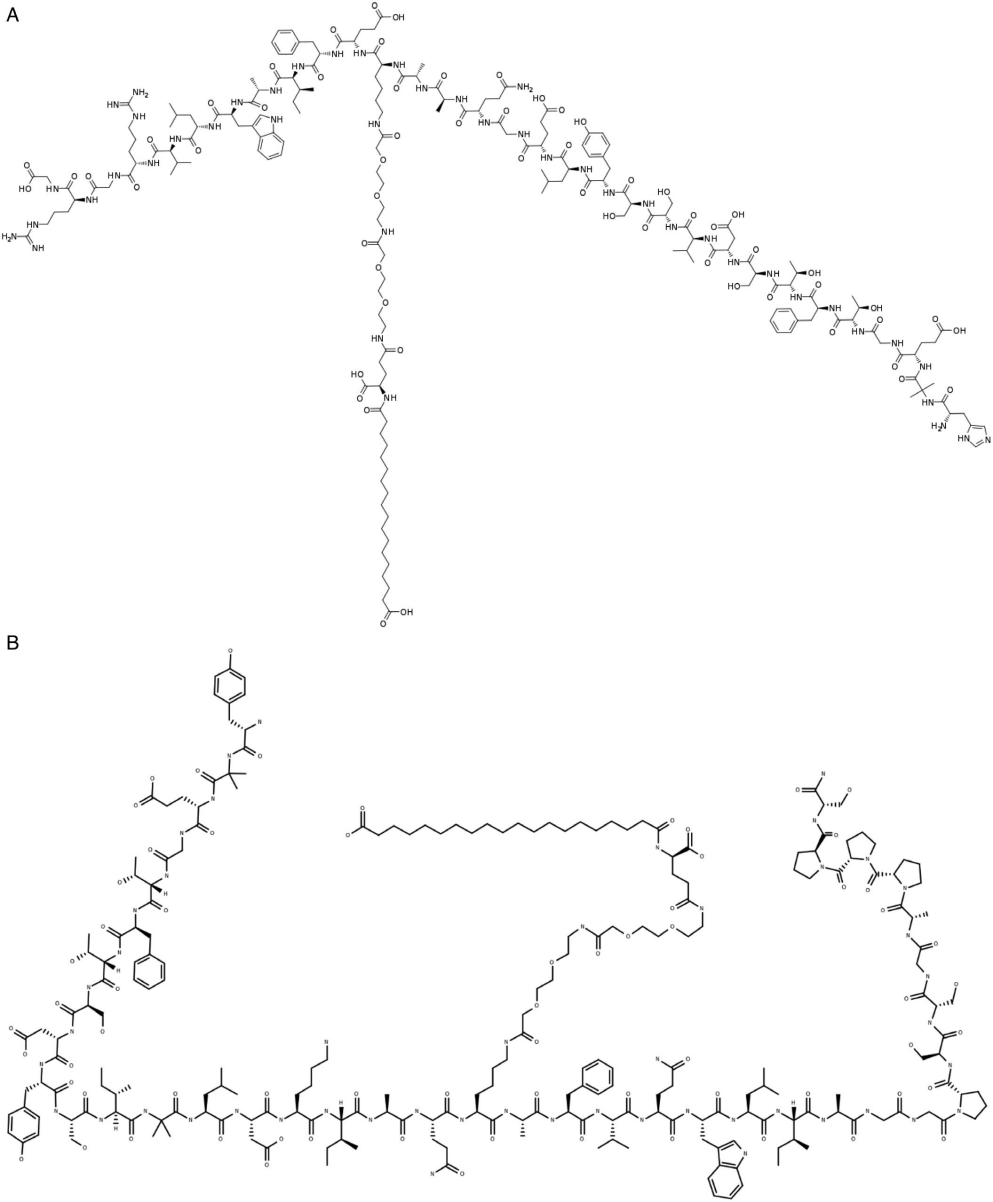
Chemical structures of Semaglutide (A), and Tirzepatide (B), recently approved anti-obesity agents.

CA VA/B inhibition on the other hand might be beneficial for the manegement of other diseases for which few therapeutic options are available. For example, several groups^74^^,^^75^ demonstrated that the elevated glucose-induced mitochondrial respiration and formation of reactive oxygen species (ROS), typical of type II diabetes, has been reversed by pharmacological inhibition of mitochondrial CAs with topiramate, in a mouse cerebral pericytes model of the disease. The connection between type II diabetes and obesity is even closer, considering that two of the recently approved anti-obesity agents, semaglutide and tirzepatide^76^ – Figure 9, were initially licenced for the manegement of type II diabetes.

Both drugs act like human glucagon-like peptide-1 (GLP-1) mimics, increasing insulin secretion and sugar metabolism. GLP-1 and the glucose-dependent insulinotropic polypeptide (GIP) are hormones involved in blood sugar control. A thought-provoking idea would be to combine this type of novel anti-obesity agents with CA VA/VB inhibitors, either as a combination therapy or by including CA inhibitory moieties in the oligopeptide structure, in order to obtain hybrids with dual action on GLP-1 as well as the two mitochondrial CA enzymes.

Overall, the field of antiobesity agents based on mitochondrial CA inhibitors achieved a certain level of maturity in the last decade, although many critical issues remain to be solved, among which more detailed pharmacological studies of the potent inhibitors already detected, a simpler method to screen compounds that interfere with the mitochondrial metabolic fluxes than the wired mitochondria one mentioned here, and last but not least, the conservatory and old fashioned idea of some scientists that CAIs are “boring drugs” that have many side effects and should not be investigated for other applications than as diuretics. Probably in the 80 s this could be even understood, as few sulphonamide pan-inhibitors CAIs were known, but the progress over that last two decades in the field^7^ demonstrated that there are many additional CA inhibition mechanisms and a wealth of new inhibitory chemotypes which are devoid of the many side effects of first and second generation such drugs.

## References

[1] Müller TD, Blüher M, Tschöp MH, DiMarchi RD. Anti-obesity drug discovery: advances and challenges. Nat Rev Drug Discov 2022;21:201–23. 34815532 10.1038/s41573-021-00337-8 PMC8609996

[2] Batchuluun B, Pinkosky SL, Steinberg GR. Lipogenesis inhibitors: therapeutic opportunities and challenges. Nat Rev Drug Discov 2022;21:283–305. 35031766 10.1038/s41573-021-00367-2 PMC8758994

[3] Burki T. European Commission classifies obesity as a chronic disease. Lancet Diabetes Endocrinol 2021;9:418. 34087168 10.1016/S2213-8587(21)00145-5

[4] de Luis D, Primo D, Izaola O, Aller R. A pilot study of gene expression analysis in peripheral blood mononuclear cells in response to a hypocaloric mediterranean diet. Dis Markers 2022;2022:3706753. 35059043 10.1155/2022/3706753 PMC8766194

[5] Redmond IP, Shukla AP, Aronne LJ. Use of weight loss medications in patients after bariatric surgery. Curr Obes Rep 2021;10:81–9. 33492629 10.1007/s13679-021-00425-1

[6] Czepiel KS, Perez NP, Campoverde Reyes KJ, et al. Pharmacotherapy for the treatment of overweight and obesity in children, adolescents, and young adults in a large health system in the US. Front Endocrinol (Lausanne) 2020;11:290. 32477270 10.3389/fendo.2020.00290 PMC7237714

[7] Supuran CT. Emerging role of carbonic anhydrase inhibitors. Clin Sci 2021;135:1233–49. 10.1042/CS20210040 34013961

[8] De Simone G, Supuran CT. Antiobesity carbonic anhydrase inhibitors. Curr Top Med Chem 2007;7:879–84. 17504132 10.2174/156802607780636762

[9] De Simone G, Di Fiore A, Supuran CT. Are carbonic anhydrase inhibitors suitable for obtaining antiobesity drugs? Curr Pharm Des 2008;14:655–60. 18336311 10.2174/138161208783877820

[10] Supuran CT, Di Fiore A, De Simone G. Carbonic anhydrase inhibitors as emerging drugs for the treatment of obesity. Expert Opin Emerg Drugs 2008;13:383–92. 18537527 10.1517/14728214.13.2.383

[11] Supuran CT. Carbonic anhydrase inhibitors as emerging drugs for the treatment of obesity. Expert Opin Emerg Drugs 2012;17:11–5. 22335448 10.1517/14728214.2012.664132

[12] Scozzafava A, Supuran CT, Carta F. Antiobesity carbonic anhydrase inhibitors: a literature and patent review. Expert Opin Ther Pat 2013;23:725–35. 23607332 10.1517/13543776.2013.790957

[13] Supuran CT. Carbonic anhydrases: novel therapeutic applications for inhibitors and activators. Nat Rev Drug Discov 2008;7:168–81. 18167490 10.1038/nrd2467

[14] Supuran CT. Advances in structure-based drug discovery of carbonic anhydrase inhibitors. Expert Opin Drug Discov 2017;12:61–88. 27783541 10.1080/17460441.2017.1253677

[15] Aspatwar A, Tolvanen MEE, Barker H, et al. Carbonic anhydrases in metazoan model organisms: molecules, mechanisms, and physiology. Physiol Rev 2022;102:1327–83. 35166161 10.1152/physrev.00018.2021

[16] Supuran CT. Carbonic anhydrases and metabolism. Metabolites 2018;8:25. 10.3390/metabo8020025 PMC6027401 29561812

[17] a) Angeli A, Carta F, Nocentini A, et al. Carbonic anhydrase inhibitors targeting metabolism and tumor microenvironment. Metabolites 2020;10:412. 10.3390/metabo10100412 PMC7602163 33066524

[18] Supuran CT. Experimental carbonic anhydrase inhibitors for the treatment of hypoxic tumors. J Exp Pharmacol 2020;12:603–17. 33364855 10.2147/JEP.S265620 PMC7751321

[19] Nishimori I, Vullo D, Innocenti A, et al. Carbonic anhydrase inhibitors. The mitochondrial isozyme VB as a new target for sulfonamide and sulfamate inhibitors. J Med Chem 2005;48:7860–6. 16302824 10.1021/jm050483n

[20] Bernardino RL, Dias TR, Moreira BP, et al. Carbonic anhydrases are involved in mitochondrial biogenesis and control the production of lactate by human Sertoli cells. Febs J 2019;286:1393–406. 30724485 10.1111/febs.14779

[21] Lynch CJ, Fox H, Hazen SA, et al. Role of hepatic carbonic anhydrase in de novo lipogenesis. Biochem J 1995;310:197–202. 7646445 10.1042/bj3100197 PMC1135873

[22] Chegwidden WR, Spencer IM. Carbonic anhydrase provides bicarbonate for de novo lipogenesis in the locus. Comp Biochem Physiol 1996;115:247–54.

[23] Hazen SA, Waheed A, Sly WS, et al. Differentiation-dependent expression of CA V and the role of carbonic anhydrase isozymes in pyruvate carboxylation in adipocytes. Faseb J 1996;10:481–90. 8647347 10.1096/fasebj.10.4.8647347

[24] Atwood PV. The structure and mechanism of action of pyruvate carboxylase. Int J Biochem Cell Biol 1995;27:231–49. 7780827 10.1016/1357-2725(94)00087-r

[25] Alldred JB, Reilly KE. Short-term regulation of acetyl CoA carboxylase in tissues of higher animals. Prog Lipid Res 1997;35:371–85. 10.1016/s0163-7827(96)00010-0 9246356

[26] Arechederra RL, Waheed A, Sly WS, et al. Effect of sulfonamides as carbonic anhydrase VA and VB inhibitors on mitochondrial metabolic energy conversion. Bioorg Med Chem 2013;21:1544–8. 22854196 10.1016/j.bmc.2012.06.053

[27] Antel J, Hebebrand J. Weight-reducing side effects of the antiepileptic agents topiramate and zonisamide. Handb Exp Pharmacol 2012;209:433–66. 10.1007/978-3-642-24716-3_20 22249827

[28] Alver A, Uçar F, Keha EE, et al. Effects of leptin and insulin on CA III expression in rat adipose tissue. J Enzyme Inhib Med Chem 2004;19:279–81. 15500001 10.1080/14756360410001720445

[29] Yamamoto H, Uramaru N, Kawashima A, Higuchi T. Carbonic anhydrase 3 increases during liver adipogenesis even in pre-obesity, and its inhibitors reduce liver adipose accumulation. FEBS Open Bio 2022;12:827–34. 10.1002/2211-5463.13376 PMC8972057 35108454

[30] Liu D, Wong CC, Zhou Y, et al. Squalene epoxidase induces nonalcoholic steatohepatitis via binding to carbonic anhydrase III and is a therapeutic target. Gastroenterology 2021;160:2467–82.e3. 33647280 10.1053/j.gastro.2021.02.051

[31] Renner SW, Walker LM, Forsberg LJ, et al. Carbonic anhydrase III (Car3) is not required for fatty acid synthesis and does not protect against high-fat diet induced obesity in mice. PLoS One 2017;12:e0176502. 28437447 10.1371/journal.pone.0176502 PMC5402959

[32] Nishimori I, Minakuchi T, Onishi S, et al. Carbonic anhydrase inhibitors. Cloning, characterization and inhibition studies of the cytosolic isozyme III with anions. J Enzyme Inhib Med Chem 2009;24:70–6. 18618322 10.1080/14756360801907143

[33] a) Supuran CT. Exploring the multiple binding modes of inhibitors to carbonic anhydrases for novel drug discovery. Expert Opin Drug Discov 2020;15:671–86. 32208982 10.1080/17460441.2020.1743676

[34] Supuran CT, Capasso C. Antibacterial carbonic anhydrase inhibitors: an update on the recent literature. Expert Opin Ther Pat 2020;30:963–82. 32806966 10.1080/13543776.2020.1811853

[35] Mincione F, Nocentini A, Supuran CT. Advances in the discovery of novel agents for the treatment of glaucoma. Expert Opin Drug Discov 2021;16:1209–25. 33914670 10.1080/17460441.2021.1922384

[36] Shank RP, Gardocki JF, Vaught JL, et al. Topiramate: preclinical evaluation of structurally novel anticonvulsant. Epilepsia 1994;35:450–60. 8156972 10.1111/j.1528-1157.1994.tb02459.x

[37] Zareba G. Zonisamide: review of its use in epilepsy therapy. Drug Today 2005;41:589–97. 10.1358/dot.2005.41.9.921095 16341290

[38] Picard F, Deshaies Y, Lalonde J, et al. Topiramate reduces energy and fat gains in lean (Fa/?) and obese (fa/fa) Zucker rats. Obesity Res 2000;8:656–63. 10.1038/oby.2000.84 11225714

[39] Klein KM, Theisen F, Knake S, et al. Topiramate, nutrition and weight change: a prospective study. J Neurol Neurosurg Psychiatry 2008;79:590–3. 18077476 10.1136/jnnp.2007.136929

[40] Gadde KM, Allison DB, Ryan DH, et al. Effects of low-dose, controlled-release, phentermine plus topiramate combination on weight and associated comorbidities in overweight and obese adults (CONQUER): a randomised, placebo-controlled, phase 3 trial. Lancet 2011;377:1341–52. 21481449 10.1016/S0140-6736(11)60205-5

[41] Garvey WT, Ryan DH, Look M, et al. Two-year sustained weight loss and metabolic benefits with controlled-release phentermine/topiramate in obese and overweight adults (SEQUEL): a randomized, placebo-controlled, phase 3 extension study. Am J Clin Nutr 2012;95:297–308. 22158731 10.3945/ajcn.111.024927 PMC3260065

[42] Gadde KM, Franciscy DM, Wagner HR, 2nd, Krishnan KR. Zonisamide for weight loss in obese adults: a randomized controlled trial. JAMA 2003;289:1820–5. 12684361 10.1001/jama.289.14.1820

[43] Gordon A, Price LH. Mood stabilization and weight loss with topiramate. Am J Psychiatry 1999;156:968–9. 10.1176/ajp.156.6.968a 10360144

[44] Lévy E, Agbokou C, Ferreri F, et al. Topiramate-induced weight loss in schizophrenia: a retrospective case series study. Can J Clin Pharmacol 2007;14:e234–9. 17565171

[45] Mahmood S, Booker I, Huang J, Coleman CI. Effect of topiramate on weight gain in patients receiving atypical antipsychotic agents. J Clin Psychopharmacol 2013;33:90–4. 23277264 10.1097/JCP.0b013e31827cb2b7

[46] Schneiderhan ME, Marvin R. Is acetazolamide similar to topiramate for reversal of antipsychotic-induced weight gain? Am J Ther 2007;14:581–4. 18090883 10.1097/MJT.0b013e31813e65b7

[47] Muñoz W, Lamm A, Poppers D, Lamm S. Acetazolamide promotes decreased consumption of carbonated drinks and weight loss. Oxf Med Case Reports 2018;2018:omy081. 30397500 10.1093/omcr/omy081 PMC6208055

[48] Wallingford NM, Sinnayah P, Bymaster FP, et al. Zonisamide prevents olanzapine-associated hyperphagia, weight gain, and elevated blood glucose in rats. Neuropsychopharmacology 2008;33:2922–33. 18322467 10.1038/npp.2008.9

[49] Aronne LJ, Wadden TA, Peterson C, et al. Evaluation of phentermine and topiramate versus phentermine/topiramate extended-release in obese adults. Obesity 2013;21:2163–71. 24136928 10.1002/oby.20584

[50] Sari C, Seip RL, Umashanker D. Case Report: Off Label Utilization of Topiramate and Metformin in Patients With BMI ≥50 kg/m2 Prior to Bariatric Surgery. Front Endocrinol 2021;12:588016. 10.3389/fendo.2021.588016 PMC7947603 33716960

[51] Casini A, Antel J, Abbate F, et al. Carbonic anhydrase inhibitors: SAR and X-ray crystallographic study for the interaction of sugar sulfamates/sulfamides with isozymes I, II and IV. Bioorg Med Chem Lett 2003;13:841–5. 12617904 10.1016/s0960-894x(03)00029-5

[52] De Simone G, Di Fiore A, Menchise V, et al. Carbonic anhydrase inhibitors. Zonisamide is an effective inhibitor of the cytosolic isozyme II and mitochondrial isozyme V: solution and X-ray crystallographic studies. Bioorg Med Chem Lett 2005;15:2315–20. 15837316 10.1016/j.bmcl.2005.03.032

[53] Supuran CT. Structure and function of carbonic anhydrases. Biochem J 2016;473:2023–32. 27407171 10.1042/BCJ20160115

[54] Supuran CT. How many carbonic anhydrase inhibition mechanisms exist? J Enzyme Inhib Med Chem 2016;31:345–60. 26619898 10.3109/14756366.2015.1122001

[55] Alterio V, Monti SM, Truppo E, et al. The first example of a significant active site conformational rearrangement in a carbonic anhydrase-inhibitor adduct: the carbonic anhydrase I-topiramate complex. Org Biomol Chem 2010;8:3528–33. 20505865 10.1039/b926832d

[56] Winum JY, Temperini C, El Cheikh K, et al. Carbonic anhydrase inhibitors: clash with Ala65 as a means for designing inhibitors with low affinity for the ubiquitous isozyme II, exemplified by the crystal structure of the topiramate sulfamide analogue. J Med Chem 2006;49:7024–31. 17125255 10.1021/jm060807n

[57] Vitale RM, Pedone C, Amodeo P, et al. Carbonic anhydrase inhibitors: molecular modeling study for the interaction of zonisamide and topiramate with isozyme VA. Bioorg Med Chem 2007;15:4152–8. 17420132 10.1016/j.bmc.2007.03.070

[58] Davis RA, Innocenti A, Poulsen SA, Supuran CT. Carbonic anhydrase inhibitors. Identification of selective inhibitors of the human mitochondrial isozymes VA and VB over the cytosolic isozymes I and II from a natural product-based phenolic library. Bioorg Med Chem 2010;18:14–8. 19962903 10.1016/j.bmc.2009.11.021

[59] Gidaro MC, Alcaro F, Carradori S, et al. Eriocitrin and Apigenin as New Carbonic Anhydrase VA Inhibitors from a Virtual Screening of Calabrian Natural Products. Planta Med 2015;81:533–40. 25590364 10.1055/s-0034-1396139

[60] a) D'Ambrosio K, Carradori S, Cesa S, et al. Catechols: a new class of carbonic anhydrase inhibitors. Chem Commun (Camb) 2020;56:13033–6. 33000794 10.1039/d0cc05172a

[61] Costa G, Gidaro MC, Vullo D, et al. Active Components of Essential Oils as Anti-Obesity Potential Drugs Investigated by in Silico Techniques. J Agric Food Chem 2016;64:5295–300. 27268752 10.1021/acs.jafc.6b02004

[62] Dick BL, Patel A, McCammon JA, Cohen SM. Effect of donor atom identity on metal-binding pharmacophore coordination. J Biol Inorg Chem 2017;22:605–13. 28389830 10.1007/s00775-017-1454-3 PMC5493394

[63] Costa G, Carta F, Ambrosio FA, et al. A computer-assisted discovery of novel potential anti-obesity compounds as selective carbonic anhydrase VA inhibitors. Eur J Med Chem 2019;181:111565. 31387062 10.1016/j.ejmech.2019.111565

[64] Costa G, Artese A, Ortuso F, Alcaro S. From Homology Modeling to the Hit Identification and Drug Repurposing: A Structure-Based Approach in the Discovery of Novel Potential Anti-Obesity Compounds. Methods Mol Biol 2021;2266:263–77. 33759132 10.1007/978-1-0716-1209-5_15

[65] Mori M, Supuran CT. Acipimox inhibits human carbonic anhydrases. J Enzyme Inhib Med Chem 2022;37:672–9. 35139721 10.1080/14756366.2022.2037579 PMC8843171

[66] Scozzafava A, Menabuoni L, Mincione F, et al. Carbonic anhydrase inhibitors. Synthesis of water-soluble, topically effective, intraocular pressure-lowering aromatic/heterocyclic sulfonamides containing cationic or anionic moieties: is the tail more important than the ring? J Med Chem 1999;42:2641–50. 10411484 10.1021/jm9900523

[67] Alterio V, Di Fiore A, D'Ambrosio K, et al. Multiple binding modes of inhibitors to carbonic anhydrases: how to design specific drugs targeting 15 different isoforms? Chem Rev 2012;112:4421–68. 22607219 10.1021/cr200176r

[68] Vullo D, Franchi M, Gallori E, et al. Carbonic anhydrase inhibitors. Inhibition of mitochondrial isozyme V with aromatic and heterocyclic sulfonamides. J Med Chem 2004;47:1272–9. 14971907 10.1021/jm031057+

[69] Güzel O, Innocenti A, Scozzafava A, et al. Carbonic anhydrase inhibitors. Aromatic/heterocyclic sulfonamides incorporating phenacetyl, pyridylacetyl and thienylacetyl tails act as potent inhibitors of human mitochondrial isoforms VA and VB. Bioorg Med Chem 2009;17:4894–9. 19539481 10.1016/j.bmc.2009.06.006

[70] Smaine FZ, Pacchiano F, Rami M, et al. Carbonic anhydrase inhibitors: 2-substituted-1,3,4-thiadiazole-5-sulfamides act as powerful and selective inhibitors of the mitochondrial isozymes VA and VB over the cytosolic and membrane-associated carbonic anhydrases I, II and IV. Bioorg Med Chem Lett 2008;18:6332–5. 18990571 10.1016/j.bmcl.2008.10.093

[71] Poli G, Bozdag M, Berrino E, et al. N-aryl-N'-ureido-O-sulfamates as potent and selective inhibitors of hCA VB over hCA VA: Deciphering the binding mode of new potential agents in mitochondrial dysfunctions. Bioorg Chem 2020;100:103896. 32413627 10.1016/j.bioorg.2020.103896

[72] Maresca A, Supuran CT. (R)-/(S)-10-camphorsulfonyl-substituted aromatic/heterocyclic sulfonamides selectively inhibit mitochondrial over cytosolic carbonic anhydrases. Bioorg Med Chem Lett 2011;21:1334–7. 21300547 10.1016/j.bmcl.2011.01.050

[73] Poulsen SA, Wilkinson BL, Innocenti A, et al. Inhibition of human mitochondrial carbonic anhydrases VA and VB with para-(4-phenyltriazole-1-yl)-benzenesulfonamide derivatives. Bioorg Med Chem Lett 2008;18:4624–7. 18644716 10.1016/j.bmcl.2008.07.010

[74] Shah GN, Morofuji Y, Banks WA, Price TO. High glucose-induced mitochondrial respiration and reactive oxygen species in mouse cerebral pericytes is reversed by pharmacological inhibition of mitochondrial carbonic anhydrases: Implications for cerebral microvascular disease in diabetes. Biochem Biophys Res Commun 2013;440:354–8. 24076121 10.1016/j.bbrc.2013.09.086 PMC3875343

[75] Salameh TS, Mortell WG, Logsdon AF, et al. Disruption of the hippocampal and hypothalamic blood-brain barrier in a diet-induced obese model of type II diabetes: prevention and treatment by the mitochondrial carbonic anhydrase inhibitor, topiramate. Fluids Barriers CNS 2019;16:1. 30616618 10.1186/s12987-018-0121-6 PMC6323732

[76] Mullard A. New hope for anti-obesity drugs. Nat Rev Drug Discov 2021;20:575. 34127838 10.1038/d41573-021-00109-4

